# Risk of Total Ankle Arthroplasty or Ankle Fusion Following Distal Tibial Fractures: A Systematic Review and Meta-Analysis

**DOI:** 10.3390/jfmk11010079

**Published:** 2026-02-16

**Authors:** Tommaso Greco, Chiara Comisi, Antonio Mascio, Federico Moretti, Virginia Cinelli, Francesco Farine, Victor Valderrabano, Giulio Maccauro, Carlo Perisano

**Affiliations:** 1 Department of Orthopedics and Rheumatological Sciences, Fondazione Policlinico Universitario Agostino Gemelli IRCCS, 00136 Rome, Italy; chiara.comisi22@gmail.com (C.C.); antonio.mascio87@gmail.com (A.M.); federicomoretti2020@gmail.com (F.M.); virginiacinelli23@gmail.com (V.C.); francesco.farine01@icatt.it (F.F.); giulio.maccauro@policlinicogemelli.it (G.M.); carlo.perisano@policlinicogemelli.it (C.P.); 2 Head-Neck and Orthopaedics Sciences, Orthopaedics and Trauma Surgery Unit, Department of Ageing, Neurosciences, Fondazione Policlinico Universitario Agostino Gemelli IRCCS, 00136 Rome, Italy; 3 Department of Life Sciences, Health, and Healthcare Professions, Link Campus University, 00165 Rome, Italy; 4 SWISS ORTHO CENTER, Schmerzklinik Basel, Hirschgässlein 15, 4051 Basel, Switzerland; vvalderrabano@swissmedical.net

**Keywords:** distal tibial fractures, tibial pilon fractures, tibial plafond fractures, total ankle replacement, total ankle arthroplasty, post-traumatic osteoarthritis

## Abstract

**Background**: Distal tibial fractures (DTFs) are a major cause of post-traumatic osteoarthritis (PTOA). The risk of conversion to total ankle arthroplasty (TAA) or ankle fusion (AF) after DTFs remains unclear, and the current literature provides heterogeneous and often incomplete data. The aim of this systematic review was to evaluate the incidence of TAA and AF following DTF-related PTOA and to explore potential predictors of conversion, including initial treatment strategy. **Methods**: A systematic review was conducted according to PRISMA guidelines. The PICO framework was applied during the study design and literature search phase to define the research question and eligibility criteria. Studies reporting adult patients with a history of DTFs who later developed PTOA and underwent TAA or AF were included. Descriptive statistics were performed. Study-level proportions of conversion to TAA, AF, or both were analyzed using random-effects meta-analysis with logit transformation. **Results**: Eight studies comprising 190,383 fractures met the inclusion criteria. Overall, 31,269 patients underwent TAA or AF, corresponding to a conversion rate of 16.4%. The pooled conversion incidence from the random-effects model was 5.6%, with considerable heterogeneity (I^2^ ≈ 100%). When procedures were analyzed separately, the pooled incidence was 0.25% for TAA and 0.76% for AF. **Conclusions**: The risk of conversion to TAA or AF after DTFs appears to be relatively low, despite the high prevalence of PTOA. The higher conversion rate observed in surgically treated fractures likely reflects the complexity of the initial fracture rather than the failure of surgical management itself. Level IV, systematic review of retrospective studies.

## 1. Introduction

Distal tibial fractures (DTFs), particularly those involving the articular surface, known as tibial pilon or tibial plafond fractures [1], represent complex and challenging injuries for orthopedic surgeons due to their intra-articular nature and high risk of complications. These fractures account for approximately 1% of all lower limb fractures, 3% to 10% of all tibial fractures, and about 20% to 40% of open fractures [2], often resulting from high-energy trauma [3,4].

There are two main classification systems for these fractures: the Rüedi–Allgöwer and the AO/OTA classification system [5,6]. The first system divides the fracture pattern into three types according to the degree of displacement and comminution of the articular surface [5]. The second one differentiates DTFs into A, B, and C types, representing respectively extra-articular, partial articular, and intra-articular types of fractures, with further sub-classifications [6].

The treatment of DTFs remains a challenge due to the complex anatomy of the distal tibia and the high-energy nature of these injuries. There are several treatment options such as external fixation, intramedullary nailing, or open reduction and internal fixation (ORIF) with different devices [7]. Recently, minimally invasive techniques have been developed to obtain better outcomes. The choice of the correct approach depends on fracture pattern, soft-tissue local conditions, and the patient’s functional request [8]. Even with advances in surgical techniques, long-term functional outcomes can be affected by complications such as residual articular incongruence, impaired joint biomechanics, chronic pain, infection, and joint stiffness, leading to a high risk of ankle post-traumatic osteoarthritis (PTOA) due to the articular surface damage [9]. PTOA following intra-articular ankle fractures has a reported incidence of 50% within the first four years and up to 75% within 5–12 years after injury [10].

PTOA is often disabling and may necessitate secondary surgical treatments: the main options today are ankle fusion (AF) or total ankle arthroplasty (TAA).

Despite the high reported incidence of PTOA following DTFs, the current prevalence of patients progressing to end-stage disease requiring joint-sacrificing procedures remains poorly defined. Previous studies have mainly focused on short- and mid-term functional outcomes, complication rates, or radiographic changes after surgery, while others have evaluated the outcomes of TAA or AF in heterogeneous populations with end-stage ankle PTOA [11,12]. However, a comprehensive analysis specifically evaluating the mid- and long-term risk of conversion to TAA or AF after DTFs is currently lacking in the literature.

Moreover, while AF remains the main solution for end-stage cases of ankle PTOA, particularly in young and adult patients, providing good long-term results, TAA is increasingly used as a viable alternative, demonstrating efficacy in restoring the ankle range of motion (ROM) and increasing the quality of life of patients [13].

According to the current literature, no previous systematic review and meta-analysis has specifically quantified the risk of conversion to TAA or AF following DTFs. The aim of this systematic review is (i) to evaluate the available literature on the risk of ankle PTOA following DTFs; (ii) to assess the subsequent need for secondary surgery, as well as TAA or AF; and (iii) to provide a comprehensive overview of this critical issue.

## 2. Materials and Methods

### 2.1. Search Strategy and Eligibility Criteria

**Figure 1:**
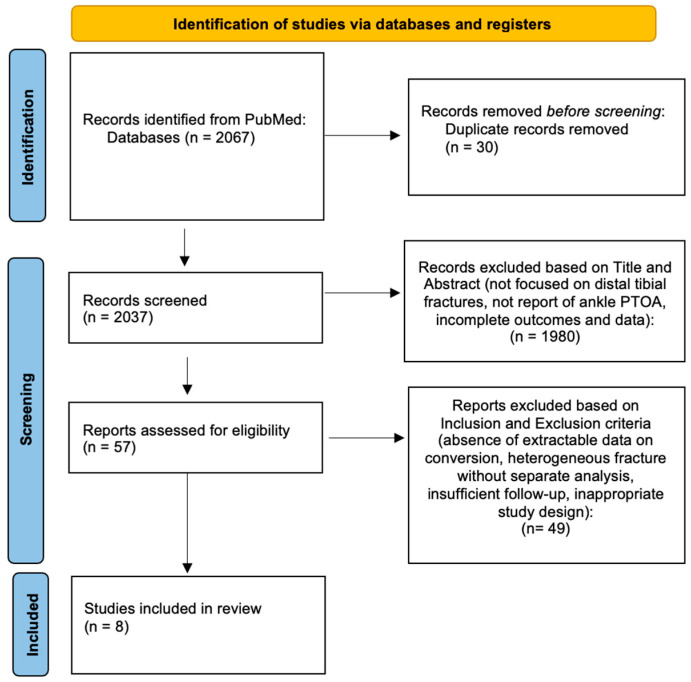
Study flowchart according to PRISMA guidelines.

According to the Preferred Reporting Items for Systematic Review and Meta-Analyses (PRISMA) guidelines [14], a systematic review of the literature was conducted from its inception up to December 2024 (Figure 1). We focused on the examination of clinical outcomes and complications in patients with DTFs. The search strategy included three online databases: MEDLINE, Web of Science, and Scopus. The keywords used, together with Boolean operators, for the research were combined as follows: (“tibial plafond fracture”) OR (“distal tibial fracture”) OR (“pilon fracture”) OR (“ankle fracture”) AND (osteoarthritis) AND (“TAA”) OR (“total ankle arthroplasty”) OR (“total ankle replacement”) OR (“TAR”) AND (“ankle fusion”) OR (“ankle arthrodesis”), and relative MeSH combinations.

To avoid overlap with other ongoing review studies, the protocol was registered online with the International Prospective Register of Systematic Reviews (PROSPERO) before submitting the review, with the registration number ID of CRD42024626111.

The PICO framework was applied to identify the key concepts for an effective search strategy, define the eligibility criteria, and improve methodological transparency and reproducibility, in accordance with recommendations for systematic reviews of observational studies [15]. In particular, the population consisted of adult patients with a diagnosis of DTFs; the exposure was the development of ankle PTOA; the comparison was surgical treatment (ST) versus non-surgical treatment (NST) as initial fracture management when available; and the outcomes were conversion to TAA or AF.

The inclusion criteria were: (i) patients aged 18 years or older with a history of DTFs who had developed ankle PTOA and (ii) studies with a follow-up of at least one year. Exclusion criteria were: (i) patients younger than 18 years; (ii) patients who underwent amputation; and (iii) studies in which outcomes related to DTFs could not be clearly distinguished due to concomitant orthopedic conditions.

Moreover, case reports, expert opinions, systematic reviews, letters to the editor, meta-analyses, and studies with incomplete data were excluded from this study because they were unsuitable for data pooling. Only papers published in English and with full texts available were considered for inclusion. Titles of journals and names of authors or supporting institutions were not masked at any stage. Moreover, if there was doubt, the corresponding author of the related articles was contacted to exclude any possibility of misunderstanding.

### 2.2. Study Assessment and Data Extraction

Titles and abstracts of potentially eligible studies were initially screened by two independent reviewers (C.C. and F.M.). Subsequently, full texts were carefully read and selected, based on the inclusion criteria, by other independent reviewers (V.C. and A.M.). Either doubts or discrepancies were resolved by the senior author (C.P.). The methodological quality of the included studies was assessed using the MINORS tool (Methodological Index for Non-Randomized Studies) for non-randomized studies, a validated scoring system composed of 12 items for comparative studies and 8 items for non-comparative studies, with each item scored from 0 to 2. Higher scores indicate better methodological quality [16]. Data were systematically extracted from each study, including type of study, participant demographics, sample size, type of fractures, ST or not NST for initial fractures, and outcomes and complications. Due to the heterogeneity of the analyzed studies in terms of patient samples and study designs, many of these values were either unreported or could not be correctly extrapolated. As a result, they were considered missing and excluded from the results. A third author (T.G.) independently verified the extracted data.

### 2.3. Statistical Analysis

Descriptive statistics were used to summarize the characteristics of the included studies and patients. Continuous variables were reported as means and ranges when available, whereas categorical variables were expressed as absolute numbers and percentages. For each study, we extracted the number of patients who underwent secondary surgery for PTOA, distinguishing, when reported, between TAA and AF. Cumulative incidences of secondary surgery were first calculated by dividing the number of TAA and/or AF procedures by the total number of fractures for each study and for the entire cohort. Ninety-five percent confidence intervals (95% CIs) for proportions were estimated using the Wilson method. In addition, an exploratory meta-analysis of proportions was performed. Study-specific event rates (TAA and/or AF, TAA alone, and AF alone) were transformed using the logit transformation, applying a continuity correction of 0.5 in studies with zero events. Random-effects models with DerSimonian–Laird estimation of the between-study variance (τ^2^) were used a priori, given the expected clinical and methodological heterogeneity among studies. Pooled proportions and their 95% CIs were then back-transformed to the original scale. Statistical heterogeneity was assessed using Cochran’s Q and quantified using the I^2^ statistic, with values > 75% indicating considerable heterogeneity. For the only study reporting both ST and NST cohorts (Axelrod et al. [17]), relative risks (RRs) and 95% CIs were calculated to compare the risk of conversion to TAA, AF, and any secondary procedure (TAA or AF) between the ST and NST groups. Log-transformed RRs and their standard errors were used to derive confidence intervals according to standard formulas.

All tests were two-sided and a *p*-value < 0.05 was considered statistically significant. Statistical analyses were performed using SPSS Statistics version 29.0 (IBM Corp., Armonk, NY, USA) for descriptive and comparative analyses and R version 4.3.1 (R Foundation for Statistical Computing, Vienna, Austria) with the “metafor” package for meta-analytic procedures.

## 3. Results

### 3.1. Search and Selection Process

The study selection flowchart is reported in Figure 1. The initial literature search yielded 2067 articles and included additional articles sourced from references in the full-text papers admitted for analysis. Thirty studies had duplicated records and were subsequently removed. The remaining 2037 papers underwent screening based on titles and abstracts. Most of them were excluded at this stage because they were not focused on DTFs, did not report ankle PTOA, and/or did not include outcomes and complete data. Fifty-seven full-text articles were subsequently assessed for eligibility. Of these, forty-nine were excluded due to several reasons such as absence of extractable data on conversion to TAA or AF, inclusion of heterogeneous fracture populations without separate analysis for distal tibial fractures, insufficient follow-up, or inappropriate study design. Finally, only eight studies met all inclusion criteria and were therefore included in the study.

### 3.2. Quality Assessment of the Included Studies

Eight articles were incorporated into the study, covering a timeframe spanning from 2006 to 2024 [14,15,16,17,18,19,20,21] (Table 1).

The methodological quality of the eight included studies, assessed using the MINORS score, ranged from 12 to 17 points, indicating an overall low-to-moderate to moderate–good quality, with no studies meeting criteria for high methodological quality.

### 3.3. Demographic Data

The main characteristics of the identified studies are summarized in Table 1.

A total of 190,383 patients were included across the eight studies analyzed. The mean age at the time of the fracture was 47.7 ± 7.3 years, ranging from 35 to 66 years. Sex distribution was balanced, with 98,983 males (52%) and 91,385 females (48%), showing no relevant gender predominance in the incidence of distal tibial fractures. Open fractures accounted for a total of 4013 cases.

Regarding initial fracture management, 103,133 patients (54%) underwent surgical treatment (ST), whereas 88,266 patients (46%) were treated non-operatively (NST). Among male patients, 47,427 (47%) received surgical treatment, whereas 51,556 (53%) were treated non-operatively. Among female patients, 55,680 (61%) were managed surgically, while 36,710 (39%) were treated non-operatively.

Only one study [17] included both treatment strategies. The remaining seven studies [18,19,20,21,22,23,24] exclusively reported outcomes from surgically treated patients.

**Table 1:** Demographic characteristics and specific data of the included studies. NR (not reported); ST (surgical treatment); NST (non-surgical treatment); M (male); F (female); TAA (total ankle arthroplasty); AF (ankle fusion); / (not applicable).

First Author, Year of Publication	MINORS Score	Patients	ST	NST	M	F	Male ST	Male NST	Female ST	Female NST	Open Fractures	TAA in ST Group	TAA in NST Group	AF in ST Group	AF in NST Group	Total of TAA or AF
Axelrod et al., 2019 [17]	17	132.409	45.133	88.266	77.344	55.065	25.778	51.556	18.355	36.710	NR	70	23	254	138	485
SooHoo et al., 2009 [18]	12	57.183	57.183	0	21.157	36.026	21.157	0	36.026	0	3.940	NR	/	NR	/	30.728
Bastìas et al., 2023 [19]	12	53	53	0	45	8	45	0	8	0	21	NR	/	NR	/	10
Regan et al., 2016 [20]	15	141	141	0	69	72	69	0	72	0	7	1	/	1	/	2
Harris et al., 2006 [21]	13	79	79	0	45	34	45	0	34	0	16	NR	/	NR	/	31
Githens et al., 2021 [23]	13	79	79	0	41	38	41	0	38	0	9	NR	/	NR	/	5
Macera et al., 2018 [22]	14	378	378	0	264	114	264	0	114	0	20	0	/	8	/	/
Chong et al., 2024 [24]	13	61	61	0	28	33	28	0	33	0	0	0	/	0	/	0
Total		190.383	103.107	88.266	98.993	91.390	47.427	51.556	54.680	36.710	4.013	71	23	263	138	31.261

### 3.4. PTOA Treatment

**Figure 2:**
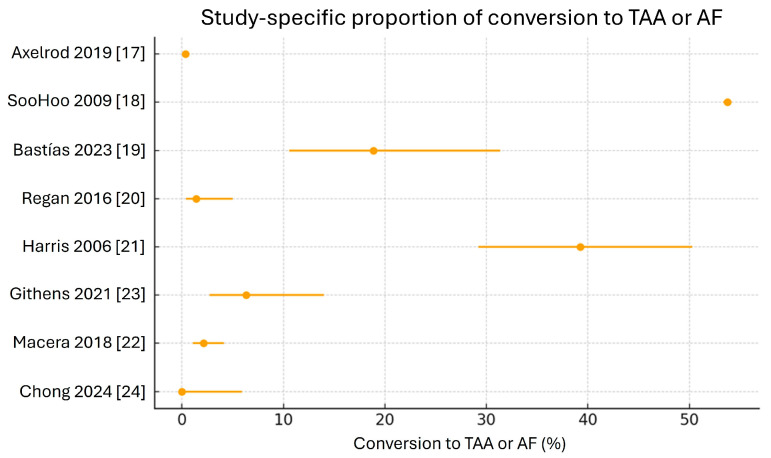
Forest plot of conversion rate (TAA: total ankle arthroplasty; AF: ankle fusion) [17,18,19,20,21,22,23,24].

Across the eight included studies, a total of 190,383 ankle fractures were analyzed. Overall, 31,269 patients underwent at least one secondary procedure for ankle PTOA (TAA or AF), corresponding to a crude cumulative incidence of 16.4% in the pooled cohort. At the study level, the proportion of patients converted to TAA or AF varied widely, ranging from 0% in the series reported by Chong et al. [24] to 53.7% in the large administrative cohort of SooHoo et al. [18]. The remaining studies showed intermediate conversion rates, from 0.4% in Axelrod et al. [17] to 39.2% in Harris et al. [21] (Figure 2).

In the random-effects meta-analysis of proportions, the pooled incidence of secondary surgery (TAA or AF) after ankle fracture was 5.6% (95% CI, 0.4–48.0%), with extremely high between-study heterogeneity (I^2^ ≈ 100%). When the type of procedure was analyzed separately, 94 TAA and 401 AF were identified in the subset of studies reporting this information in detail. The random-effects model yielded a pooled proportion of 0.25% (95% CI, 0.04–1.7%; I^2^ ≈ 76%) for TAA and 0.76% (95% CI, 0.18–3.1%; I^2^ ≈ 90%) for AF.

These estimates should be interpreted cautiously, as they are derived from a limited number of heterogeneous studies and are strongly influenced by the large administrative database study by SooHoo et al. [18], which includes a substantially higher number of patients and events compared with the other included studies. As a result, this study disproportionately contributes to the pooled incidence and largely accounts for the observed heterogeneity.

### 3.5. Comparison Between Surgically and Non-Surgically Treated Fractures

Only Axelrod et al. [17] provided separate cohorts of patients initially managed surgically (ST, *n* = 45,133) or non-surgically (NST, *n* = 88,266) after ankle fracture. In this study, 324 patients in the ST group and 161 in the NST group later underwent TAA or AF, corresponding to an absolute risk of 0.72% in the ST cohort and 0.18% in the NST cohort. The risk of any secondary procedure (TAA or AF) was therefore significantly higher in patients who had received surgical treatment at the time of the index fracture (RR = 3.94; 95% CI, 3.26–4.75). When individual procedures were considered, the relative risk of conversion to TAA was 5.95 (95% CI, 3.72–9.53), while the relative risk of AF was 3.60 (95% CI, 2.93–4.43) in surgically treated patients compared with those initially managed non-operatively.

Not all the studies reported the exact number of TAA and AF procedures separately; some provided only the total number of surgical procedures (including both TAA and AF) performed for the treatment of ankle PTOA [18,19,21,23,24].

Regarding the mean time from PTOA to secondary surgery, only the study by Axelrod et al. accurately reported these data [17]. In particular, the mean interval from the previous treatment of DTFs to TAA was 6.9 years, while the mean interval from the previous surgery to AF was 2.8 years.

## 4. Discussion

Correct management of distal tibial fractures (DTFs), particularly when involving articular displacement, remains a major challenge for orthopedic surgeons. Treatment options range from conservative measures to various surgical approaches, with the primary goal of achieving anatomic reduction, preserving soft-tissue integrity, and restoring joint congruity [25].

PTOA is one of the most frequent long-term complications after DTFs, with an incidence reported as high as 50% within four years and up to 75% over 5–12 years of follow-up [10]. Symptoms include pain, stiffness, swelling, and progressive loss of ankle range of motion, eventually leading to severe functional impairment [26].

When PTOA becomes symptomatic and refractory to conservative measures, surgical management includes both joint-preserving procedures (JPS) and joint-sacrificing strategies (JSS), the latter represented mainly by TAA and ankle fusion AF [27]. Although both procedures aim to relieve pain, they differ in biomechanical impact, functional expectations, and long-term survivorship [12].

The aim of this systematic review was to analyze the risk of conversion to TAA or AF among patients developing PTOA after DTFs. One of the strengths of this study is that it focuses on a topic that remains relatively underexplored: the long-term likelihood of requiring TAA or AF following a fracture of the distal tibia. However, the reliability and precision of the findings are inevitably influenced by the limited and heterogeneous nature of the currently available literature, which provides incomplete and inconsistent data on this outcome.

Among the eight included studies, only one [17] directly compared ST and NST fractures, while the remaining studies only reported outcomes after surgical fixation. Moreover, in four studies, the type of secondary surgery (TAA or AF) was not specified separately, further limiting the ability to accurately quantify the relative conversion rates [18,19,21,24].

Despite these limitations, the present study suggests that conversion to TAA or AF after DTFs may occur in a relatively small proportion of patients. Across all included studies, the crude cumulative conversion rate was 16.4%, although this figure was strongly influenced by one large administrative cohort. In the exploratory random-effects meta-analysis, the pooled incidence of secondary surgery was 5.6%, with substantial heterogeneity. When procedures were analyzed separately, the pooled incidences were 0.25% for TAA and 0.76% for AF, although these values must be interpreted cautiously because only a subset of the studies reported these outcomes in detail.

At the individual study level, results were heterogeneous. Regan et al. [20] reported only two cases of conversion among 141 surgically treated fractures, while Bastías et al. [19] documented ten cases among 53 patients. Conversely, Chong et al. [24] found no conversions over the entire follow-up period.

Our results overall align with these observations, showing a relatively low pooled incidence of conversion to TAA or AF despite the high prevalence of post-traumatic degenerative changes. The wide variability in reported conversion rates across individual studies likely reflects differences in fracture severity, treatment protocols, follow-up duration, and patient populations.

Sex distribution appeared balanced across studies, with males representing 52% and females representing 48% of the cohort, and no consistent differences in conversion rates were observed between genders. The mean age at the time of fracture was approximately 47.7 years, although only Axelrod et al. [17] provided detailed information on the interval between primary treatment and conversion surgery, reporting a mean time of 6.9 years for TAA and 2.8 years for AF.

A key finding emerges from the only matched-cohort analysis: surgically treated fractures were almost four times more likely to require TAA or AF compared with fractures initially managed non-operatively [17]. The risk of conversion to TAA was nearly sixfold higher, while the risk of AF was more than threefold higher in the surgical group. This difference is likely attributable to confounding by indication, because surgically treated patients typically present with more severe, displaced, or comminuted fractures—conditions strongly associated with an increased risk of developing PTOA.

### 4.1. Limitations

This review has several limitations. First, only eight studies met the inclusion criteria, and most were retrospective, with the inherent risk of selection bias, information bias, and confounding by indication. Second, a substantial proportion of studies (five out of eight; 62.5%) did not distinguish between TAA and AF, reporting only the total number of secondary procedures; this limited the precision of pooled estimates for each specific surgery. Third, key clinical variables such as fracture pattern and severity, initial displacement, type of fixation device, soft-tissue condition, and time from fracture to PTOA onset were inconsistently reported or entirely absent.

Fourth, only one study provided a direct comparison between surgically and non-surgically treated fractures, making it impossible to perform a robust meta-analytic comparison between treatment strategies. Fifth, follow-up duration varied widely among the included studies and was sometimes insufficient to capture all late conversions to TAA or AF. Finally, the marked heterogeneity in sample size, case histories, and treatment protocols strongly influenced the results.

To mitigate these limitations, strict inclusion criteria were applied, a rigorous study selection was conducted according to PRISMA guidelines, and random-effects models were used to account for the substantial clinical and methodological heterogeneity across studies.

### 4.2. Clinical Implications and Future Directions

The current results suggest that ankle PTOA is a common long-term consequence of DTFs, and only a relatively small proportion of patients ultimately require JSS such as TAA or AF. This information is relevant for patient counseling and outcome management, allowing clinicians to provide a more realistic estimation of long-term risks. Moreover, the higher conversion rates observed in the ST group likely reflect greater initial fracture severity rather than failure of surgical management, highlighting the importance of individualized treatment strategies and long-term follow-up in high-risk patients.

Future studies should focus on large, prospective cohort studies with standardized reporting of fracture characteristics, treatment strategies, and long-term outcomes. Identifying reliable predictors of progression from ankle PTOA to end-stage disease requiring JSS remains a key research priority. Longer follow-up periods and better stratification based on fracture severity and patient-related factors may help refine treatment algorithms and improve decision-making in this complex patient population.

## 5. Conclusions

This review indicates that the cumulative rate of TAA or AF was 16.4% across all included fractures, but the pooled estimate from random-effects meta-analysis suggested a lower overall incidence, with substantial between-study heterogeneity. Despite the high reported frequency of PTOA after DTFs, only a subset of patients described in the available literature progressed to JSS such as TAA or AF. However, the current evidence is limited and heterogeneous. Large, high-quality prospective and standardized studies are needed to better define the true incidence, risk factors, and optimal indications for TAAand AF in this complex patient population.

## Data Availability

No new data were created or analyzed in this study.

## Abbreviations

The following abbreviations are used in this manuscript:

AF	Ankle Fusion
DTFs	Distal Tibial Fractures
JPS	Joint-Preserving Surgery
JSS	Joint-Sacrificing Surgery
MINORS	Methodological Index for Non-Randomized Studies
NST	Non-Surgical Treatment
ORIF	Open Reduction and Internal Fixation
PRISMA	Preferred Reporting Items for Systematic Review and Meta-Analyses
PTOA	Post-Traumatic Osteoarthritis
ROM	Range of Motion
ST	Surgical Treatment
TAA	Total Ankle Arthroplasty
